# Hemangiopericytoma of Greater Omentum Presenting as a Huge Abdominal Lump

**DOI:** 10.4103/1319-3767.39626

**Published:** 2008-04

**Authors:** Damodar Chatterjee, Pradip Sarkar, Niladri Sengupta, W. Gopimohan Singh

**Affiliations:** Department of Surgery, Agartala Government Medical College and G. B. Pant Hospital, Agartala, Tripura, India

**Keywords:** Greater omentum, hemangiopericytoma, laparotomy

## Abstract

Hemangiopericytoma is a rare neoplasm that can occur in any part of the human body, but it rarely develops in the greater omentum. We report a case of a patient who presented with a huge abdominal lump. At laparotomy, a huge vascular tumor, which was observed originating from the greater omentum, was resected. Histopathology investigation revealed this tumor as a benign hemangiopericytoma with a malignant potential.

Hemangiopericytoma is a rare tumor featuring Zimmermann's pericytes, which was first described by Stout and Murry.[1] Pericytes are rudimentary cells that have contractile proteins that regulate blood flow through capillaries. Hemangiopericytoma develops in deep soft tissues, especially those of the extremities or retroperitoneum, and commonly affects middle-aged patients.[2] Development of this tumor in the greater omentum, according to the literature review, is very rare, as only 14 cases have been reported till date.[3] Tumor size is an important prognostic factor, and surgical resection provides the only chance of effective cure.

## CASE REPORT

**Figure 1:**
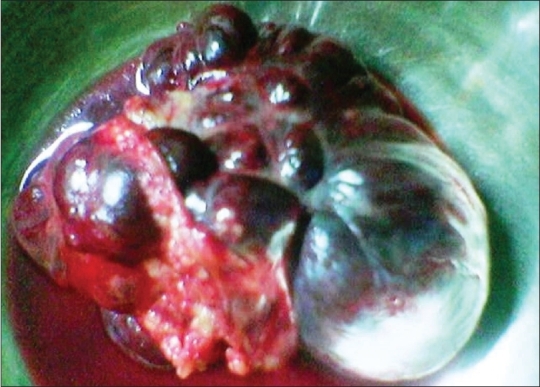
Postoperative image of resected specimen

**Figure 2:**
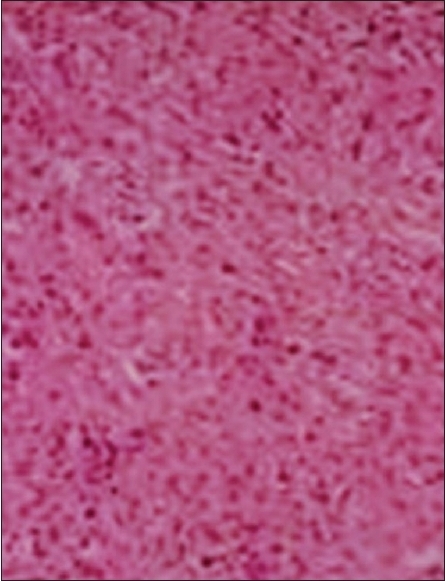
Histopathology image

A 41-year-old male patient was admitted to the with a huge abdominal lump. Abdominal ultrasonography diagnosed it as a retroperitoneal tumor. Fine needle aspiration cytology (FNAC) described it as being mesenchymal in origin. During laparotomy, a huge, dark brown, lobulated mass weighing 470 g measuring 12.5 × 8.5 × 7.5 cm^3^ was detected arising from the greater omentum [Figure 1]. This mass was resected and sent for histopathological examination, which diagnosed it as hemangiopericytoma [Figure 2]. The tumor was also positive for CD34. Recovery was uneventful and the patient was advised to visit the hospital for follow ups.

## DISCUSSION

Hemangiopericytoma is a rare tumor featuring Zimmerman's pericytes, which was first described by Stout and Murry.[1] Hemangiopericytoma represent < 1% of all vascular neoplasms.[4] This is a tumor of mesenchymal perivascular cell origin with dilated vascular spaces spread throughout the entire tumor. The tumor cells are surrounded by reticulin, and are negative for muscles, nerve sheaths and epithelial markers, and positive for CD34.[5] This tumor arising in the greater omentum is extremely rare and only a few cases (14 in number) were reported in the English literature.[6–8] Hypervascularity is a contraindication for FNAC, therefore a histopathological diagnosis was established after an excision.[4] A review of the reported cases suggests that surgical resection with subsequent radiotherapy provides the only chance of effective cure[4,9] and revealed that three patients died of recurrence. Metastases occur by hematogenous and lymphogenous routes affecting mainly the liver, bones, and regional lymph nodes. Multiple hepatic and bone metastases 12 years after tumor resection have been reported.[4] Therefore, the evaluation of malignant potential is important. Recent reports proposed that malignant hemangiopericytoma should be suspected in cases where the tumor size is >5 cm.[6] Different studies suggest that surgical resection provides the only chance of effective cure and a tumor size of ≥20 cm predicts an unfavorable prognosis.
